# Artificial intelligence and the acceleration of Alzheimer’s research - From promise to practice

**DOI:** 10.1016/j.tjpad.2025.100421

**Published:** 2025-12-01

**Authors:** Gregory J. Moore, Niranjan Bose, Husseini K. Manji, Eric M. Reiman, Reisa Sperling

**Affiliations:** a Gates Ventures, Kirkland, USA; b Alzheimer’s Disease Data Initiative, Kirkland, USA; c Oxford University, Oxford, United Kingdom; d Banner Health Alzheimer’s Institute, Tucson, USA; e Massachusetts General Hospital and Harvard Medical School, Boston, USA

Alzheimer’s disease (AD) remains one of the most formidable challenges in modern medicine. Despite extraordinary advances in molecular neuroscience, imaging, and biomarker science, therapeutic progress has been painfully slow. Drug development timelines remain long, clinical trial costs remain high, and millions of patients and families continue to face the devastating impact of a disease for which cures remain elusive. Without new solutions, the global prevalence of AD and related dementias is projected to triple in the next quarter century [1], which makes the urgency of new solutions undeniable.

In April of this year, several of us contributed to a *Nature Medicine* perspective [2] outlining a call to action: artificial intelligence (AI) offers the potential to overcome entrenched bottlenecks in AD research and accelerate the path to effective prevention and treatment. This special issue of JPAD represents the next step in that journey. Across its pages, leading scientists and clinicians from around the world illustrate how AI is already reshaping the landscape of AD research — from biomarker discovery to trial innovation — and offer a glimpse of what lies ahead.

## 1 Biomarkers and early detection

One of the clearest opportunities for AI is in the development of scalable biomarkers for early detection. By leveraging multimodal data streams — from imaging and fluid biomarkers to speech and digital phenotyping — AI can detect subtle patterns that elude conventional analyses. In this issue, Wang et al.[3] illustrate how AI-enabled speech analysis can identify prodromal cognitive decline,while Au et al.[4] propose re-envisioning the widely adopted A–T–N framework [5] to integrate digital and AI-derived measures. These contributions exemplify how the combination of machine learning and novel data modalities can shift us toward earlier, more precise detection and stratification — a critical step for prevention trials and clinical care alike. Beyond detection, AI also offers opportunities to uncover causal and modifiable risk and resilience factors — from genetics to environmental exposures — which can serve as targets for risk-reduction and resilience-building strategies in prevention trials.

## 2 Drug discovery and knowledge integration

Equally transformative is AI’s role in therapeutic discovery. The explosion of omics data, neuroimaging results, and real-world clinical observations has created a landscape that is too vast for any human researcher to navigate. AI systems can now synthesize these complex datasets into evolving models of AD biology. In this issue, Wittenberg et al. [6] describe how Big Data and AI are accelerating drug discovery pipelines, while Funk et al. [7] demonstrate how machine learning can build coherent, integrative models from noisy and even contradictory findings. Extending this vision, Roberts and Landsness et al. [8] advance the concept of an “AI biomedical scientist assistant” — a partner that augments human creativity in hypothesis generation, experimental design, and data interpretation. Collectively, these advances suggest a future in which AI not only accelerates discovery but fundamentally reshapes how we think about biomedical science.

## 3 Transforming clinical trials

Clinical trials remain among the most time- and cost-intensive aspects of AD research. Here, too, AI is beginning to make inroads. Yigamawano et al.[9] and Welchman & Kourtzi [10] describe how advanced machine learning methods can improve patient recruitment and stratification, reducing attrition and enhancing trial efficiency. Complementing these strategies, digital twin models — highlighted across multiple contributions in this issue — offer the potential to simulate disease trajectories and treatment responses before interventions are tested in vivo. In parallel, contemporaneous work outside of this special issue, such as Devanarayan et al. [11], has demonstrated that multimodal prognostic modeling of individual cognitive trajectories can substantially improve efficiency in prevention trials, with the potential to reduce required sample sizes by more than one-third. Together, these approaches point to a future in which both trial enrollment and trial durations are shorter, more predictive, and more patient centered. Together, these approaches point to a future in which both trial enrollment and trial durations are shorter, more predictive, and more patient centered.

## 4 Ethics, equity, and data sharing

The promise of AI will not be realized without attention to its risks. AI systems reflect the data on which they are trained, raising the specter of bias, inequity, and limited generalizability. Kolachalama et al. [12] remind us that reproducibility and fairness must remain central priorities, urging safeguards to prevent AI from amplifying existing disparities. Building on this, Adams et al. [13] demonstrate how large language models can be harnessed for semantic harmonization across diverse Alzheimer’s cohorts, showing that harmonization is not just a technical advance but a cornerstone of equitable and scalable data use. More broadly, the contributions in this issue emphasize that success will depend on data-sharing frameworks that are both privacy-preserving and globally inclusive. Without such frameworks — and the diverse datasets and international collaboration they enable — AI-driven tools risk serving only a fraction of the world’s patients and face the peril of overlooking critical insights from global populations.

## 5 Looking ahead

The contributions in this special issue reflect both momentum and responsibility. The momentum is evident in the rapid emergence of AI applications across biomarker science, therapeutic discovery, and clinical research. The responsibility lies in ensuring that these advances are rigorously validated, ethically grounded, and equitably distributed.

The convergence of AI and neuroscience represents a pivotal opportunity to redefine how we study, diagnose, and ultimately treat Alzheimer’s disease. Realizing this potential will also require careful attention to how these tools are integrated into clinical practice — from interoperability with health records to clinician and patient engagement, and the development of appropriate regulatory frameworks. AI is not an autonomous solution, but rather a powerful amplifier and accelerator of human expertise. Its greatest value will come from fusing computational power with the insight, creativity, and compassion of the scientific and medical community.

This issue of JPAD articulates the first wave of AI’s impact in AD research. It also makes plain the imperative: to scale these approaches, sustain them across global contexts, and ensure they address the urgent needs of patients and families. With shared purpose and deliberate action, the field can seize this opportunity to move from incremental progress to transformative change — and ultimately to a future in which Alzheimer’s is preventable and curable.

## Declaration of generative AI and AI-assisted technologies in the writing process

The manuscript was originally drafted without the use of AI technologies; and subsequently AI (ChatGPT) was used to improve readability and formatting of the manuscript and its associated references. Subsequently the authors reviewed and edited the content as needed and take full responsibility for the content of the publication.

## CRediT authorship contribution statement

**Gregory J. Moore:** Conceptualization, Writing – original draft. **Niranjan Bose:** Writing – review & editing. **Husseini K. Manji:** Writing – review & editing. **Eric M. Reiman:** Writing – review & editing. **Reisa Sperling:** Writing – review & editing.

## Declaration of competing interest

The authors declare the following financial interests/personal relationships which may be considered as potential competing interests: Gregory J. Moore MD, PhD reports financial support was provided by Gates Ventures. Gregory J. Moore MD, PhD reports a relationship with Gates Ventures that includes: consulting or advisory. If there are other authors, they declare that they have no known competing financial interests or personal relationships that could have appeared to influence the work reported in this paper.
